# Disentangling environmental drivers of circadian metabolism in desert-adapted mice

**DOI:** 10.1242/jeb.242529

**Published:** 2021-09-27

**Authors:** Jocelyn P. Colella, Danielle M. Blumstein, Matthew D. MacManes

**Affiliations:** University of New Hampshire, Department of Molecular, Cellular, and Biomedical Sciences, Durham, NH 03824, USA

**Keywords:** Circadian torpor, Deer mice, Lipogenesis, Metabolism, *Peromyscus*, Physiology

## Abstract

Metabolism is a complex phenotype shaped by natural environmental rhythms, as well as behavioral, morphological and physiological adaptations. Metabolism has been historically studied under constant environmental conditions, but new methods of continuous metabolic phenotyping now offer a window into organismal responses to dynamic environments, and enable identification of abiotic controls and the timing of physiological responses relative to environmental change. We used indirect calorimetry to characterize metabolic phenotypes of the desert-adapted cactus mouse (*Peromyscus eremicus*) in response to variable environmental conditions that mimic their native environment versus those recorded under constant warm and constant cool conditions, with a constant photoperiod and full access to resources. We found significant sexual dimorphism, with males being more prone to dehydration than females. Under circadian environmental variation, most metabolic shifts occurred prior to physical environmental change and the timing was disrupted under both constant treatments. The ratio of CO_2_ produced to O_2_ consumed (the respiratory quotient) reached greater than 1.0 only during the light phase under diurnally variable conditions, a pattern that strongly suggests that lipogenesis contributes to the production of energy and endogenous water. Our results are consistent with historical descriptions of circadian torpor in this species (torpid by day, active by night), but reject the hypothesis that torpor is initiated by food restriction or negative water balance.

## Abstract (precis)

**Summary:** Continuous metabolic phenotyping of desert-adapted cactus mice (*Peromyscus eremicus*) identifies significant metabolic differences between sexes and circadian patterning consistent with lipogenesis and environmental entrainment.

## INTRODUCTION

Our planet is undergoing unprecedented environmental change, which is exerting new, strong selective pressures on species, forcing them to relocate, adapt *in situ* or risk extinction. Warming temperatures and increasing desertification are predicted to impact most species in North America (Parmesan and Yohe, 2003; Urban, 2015; IPCC, 2018). One productive line of research relates to desert animals (Kordonowy et al., 2017; MacManes, 2017; Riddell et al., 2019, 2021; Rocha et al., 2021), which are already adapted to hot and dry conditions and therefore may exhibit mechanisms (e.g. molecular, behavioral or physiological) through which animals could adapt to a warming climate (Vale and Brito, 2015; Rocha et al., 2021). Much research has been devoted to understanding genetic correlates with heat and dehydration tolerance in extant desert-adapted taxa. Of equal importance are the physiological adaptations of desert species, which are also predicted to play an important role in species’ responses to climate change (Kearney et al., 2009; Boyles et al., 2011; Huey et al., 2012). Adaptive physiological mechanisms do not evolve under stable environmental conditions, as they have traditionally been measured (Hulbert and Else, 2004). Instead, water economies and thermoregulation are shaped by natural environmental rhythms, including circadian and seasonal variation in temperature, humidity and photoperiod, among other variables (Morrison et al., 2008; Seebacher, 2009). Circadian rhythms are observed in most mammals and are in part governed by genetics and the interplay between the perception of environmental change and neuroendocrine-mediated physiological responses (Miranda-Anaya et al., 2019). Examination of the physiological responses of organisms to both stable and dynamic environmental conditions provides a window into the flexibility of such responses to changing environments and will be increasingly important for understanding and ultimately predicting species’ responses to climate change (Garland and Carter, 1994; Dalziel et al., 2009; Easterling et al., 2000; IPCC, 2012, 2018; Huey et al., 2012).

Deer mice of the genus *Peromyscus* inhabit an exceptional range of thermal environments in North America, from cold high-elevation mountain tops to hot arid deserts (Hock, 1964; Cheviron et al., 2014; MacManes and Eisen, 2014; Harris and Munshi-South, 2017; Storz et al., 2019). Under projected climate scenarios (e.g. IPCC, 2018), adaptations that increase fitness in warmer, drier environments are predicted to be increasingly favored (Merkt and Taylor, 1994; Gienapp et al., 2008; Kingsolver, 2009). Cactus mice (*Peromyscus eremicus*) are endemic to the southwestern deserts of North America and, like many desert taxa, exhibit a number of morphological (large ears, large surface to volume ratio), behavioral (saliva spreading: Murie, 1961; nocturnality: Veal and Caire, 1979), and physiological (low body temperature: McNab and Morrison, 1963; anuria: Kordonowy et al., 2017) adaptations to accommodate high-heat, low-water desert environments. Further, the suitability of this species to life in captivity also makes them a promising experimental model for identifying and characterizing the abiotic factors (e.g. temperature, humidity, photoperiod, diet, resource availability, etc.) that shape adaptive physiological responses.

Desert species, including cactus mice, can withstand environmental extremes, but are also adapted to large circadian fluctuations in environmental conditions, most notably temperature, humidity and water availability. In the Sonoran Desert, daily temperatures can range between −4 and 38°C (Reid, 1987; Sheppard et al., 2002), with weeks or months passing between rainfall events. Water is required for basic physiological function (Häussinger, 1996; Montain et al., 1999), but water loss often exceeds intake in desert environments (Heimeier et al., 2002). Pulmocutaneous evaporation is the primary mechanism of water loss in *Peromyscus*, when at rest, and this loss is further exacerbated by extreme heat and aridity (MacMillen, 1983; Cheuvront et al., 2010). Thermal tolerance in *P. eremicus* differs between sexes. When subjected to high temperatures (upper critical limit, 38°C), all males expired, while females survived (McNab and Morrison, 1963). In other mammals, exposure to high heat, just under an organism's critical limit, disproportionately impacts female reproductive success and fitness over that of males, as viable spermatozoa can be quickly regenerated, while the damage or loss of an embryo is irreversible (Moore, 1926). Such sex-specific selection also shapes traits that are not directly associated with reproductive function, including growth rate, thermoregulation, metabolic biorhythms and environmental sensing (Glucksmann, 1974; McPherson and Chenoweth, 2012; Juárez-Tapia and Miranda-Anaya, 2017; Calisi et al., 2018). Sexual dimorphism in metabolism, body size and composition, and heat tolerance is observed in other mammals, including humans (Glucksmann, 1974; Mittendorfer, 2005; Pomatto et al., 2018), and is expected in *P. eremicus*, but the degree and direction of differentiation remain unknown.

Most endotherms are limited in their ability to metabolically respond to suboptimal environmental conditions (Bennett and Ruben, 1979; Fuglei and Øritsland, 1999), with a notable exception being those that can enter torpor (Ruf and Geiser, 2015), including cactus mice and other *Peromyscus* species. In response to water stress, cactus mice enter seasonal torpor, reducing above-ground activity and depressing metabolism to conserve endogenous resources during the dry season (Macmillen, 1965, 1983). Early investigations found that torpor in cactus mice could be initiated at any time by food restriction, but could only be triggered by dehydration during the summer months, which suggests some degree of seasonal metabolic entrainment (Macmillen, 1965, 1972). As a nocturnal species, cactus mice reduce activity during the light phase, seeking cooler, shaded parts of their habitat, hiding underground or even entering circadian torpor (torpid by day, active by night), presumably to avoid excess evaporative water loss in a water-limited environment. The role of photoperiod and ambient temperature in mediating these adaptive responses remains to be explored (MacMillen, 1965, 1972, 1983; Degen, 2012; Alonso et al., 2016). Ultimately, a balance exists between environmental heterogeneity (e.g. resource availability, thermal stress), which requires animals to sense and respond to external conditions in real time through reactionary mechanisms, and environmental predictability, which can lead to the evolution of anticipatory biorhythms based on regular periodicity of abiotic conditions (e.g. photoperiod, daily thermal variation).

Here, we examined patterns of metabolic variation in male and female cactus mice across a 24 h period under diurnally variable temperature and humidity to understand how desert species accommodate environmental variation. We contrast these results with measurements taken under constant temperature and humidity to determine whether traditional investigations of metabolism that use constant conditions provide an accurate picture of an organism's metabolic physiology. By manipulating temperature and humidity, while maintaining a constant photoperiod and access to food and water, we tested the role of temperature, humidity, photoperiod and resource availability on the governance and timing of circadian torpor and adaptive metabolism in this species. We hypothesized that cactus mouse metabolism will be well tuned to circadian patterns of environmental variation, and that circadian patterning will be disrupted under constant treatments. Comparing metabolic responses between sexes and across constant and variable environments, we show that (i) males are more prone to dehydration than females, (ii) patterns of metabolic variation are well tuned to environmental cycling, and (iii) lipogenesis offers a potential mechanism of endogenous water production, employed only under variable environmental conditions.

## MATERIALS AND METHODS

### Mice and environmental conditions

Mice were cared for, handled and sampled in accordance with the University of New Hampshire's Institutional Animal Care and Use Committee (AUP #180705). All mice used in this study were bred from wild-derived lines maintained at the *Peromyscus* Genetic Stock Center of the University of South Carolina (Columbia, SC, USA) originally collected in Tucson, AZ, USA, in 1993 (∼30–50 generations ago). *Peromyscus* live for 4–5 years in captivity and remain reproductive for at least 2 years (Davis and Keisler, 2016), and captive populations maintain high levels of genetic polymorphism (Joyner et al., 1998). The University of New Hampshire colony was founded in 2013 from 40 mice and supplemented by 30 more *Peromyscus* Genetic Stock Center individuals in 2018. The colony does not exhibit molecular or physiological evidence of extreme inbreeding and all individuals have the opportunity to reproduce, indicating the colony well represents *P. eremicus* populations from the American southwest. All mice were sexually mature, non-reproductive adults between 3 and 9 months of age. Animals were fed standard LabDiet 5015* [19% protein, 26% fat, 54% carbohydrates; food quotient (FQ) of 0.89; moisture content ≤12%] and provided with water *ad libitum* from a well-sealed water bottle. Unlimited access to both water and food throughout all experiments eliminates the effect of variable levels of pre-formed water in food. Body mass (g) was collected for each animal at the start of each experiment. Mice were housed alone, with bedding, and acclimated to experimental cages and conditions for 24 h prior to measurement.

**Fig. 1.**
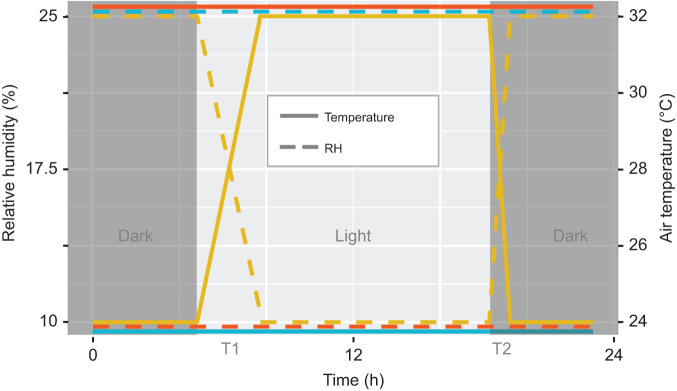
**Experimental conditions across a 24** **h cycle for each cactus mouse (*Peromyscus eremicus*) treatment group.** Diurnally variable treatment (yellow), constant warm treatment (orange) and constant cool treatment (blue). Temperature is indicated by a solid line and relative humidity (RH) by dashed lines. Light and dark photoperiod are indicated by light and dark gray boxes, respectively. T1, morning transition; T2, evening transition.

Environmental conditions for each treatment are illustrated in Fig. 1. In brief, the desert simulation chamber has a photoperiodic cycle of 16 h of light and 8 h of darkness, with transitions occurring at 06:00 h (dark to light) and 20:00 h (light to dark) to mimic sunrise and sunset. Photoperiod was constant across all treatments. Prior to treatment, all animals were subject to circadian environmental variation to mirror the natural conditions of southwestern deserts. Under circadian or diurnally variable environmental conditions, the chamber (which houses all animal chambers) was held at a temperature of 32°C and relative humidity (RH) of 10% during the light phase, from 09:00 h to 20:00 h. Changes to the desert simulation chamber environment (e.g. temperature, humidity) were then reflected in the un-sealed animal and empty baseline chambers. We used flow-through respirometry to record temperature and humidity continuously throughout the experiments using a Sable Systems International (SSI, Las Vegas, NV, USA) Field Metabolic System (FMS). At 20:00 h, chamber temperature was decreased and humidity was gradually increased over the course of 1 h to 24°C and 25% RH. This 1 h transition period (20:00–21:00 h) is hereafter referred to as the evening transition (T2), whereby the room shifts from warm with low humidity to cooler and more humid, following lights-out at 20:00 h. After 21:00 h, conditions were held constant throughout the dark phase until 06:00 h. At 06:00 h, the light phase was initiated and there was a gradual 3 h transition from cool temperatures and higher humidity (24°C and 25% RH) to warmer temperatures and lower humidity (32°C and 10% RH), during what is hereafter referred to as the morning transition period (T1). Temperatures were selected to mimic normal diurnal fluctuations of the Sonoran Desert, which typically change by 10–20°C between day and night. We tested two constant temperature treatments: a constant warm treatment that maintained light phase temperature and humidity (32°C and 10% RH) levels throughout the entire experiment and a constant cool treatment, which maintained dark phase environmental conditions (24°C and 25% RH) throughout. Lower critical temperature is estimated at 29°C and upper critical temperature at 35–38°C (McNab and Morrison, 1963); therefore, thermoneutrality for *P. eremicus* spans 29–35°C for desert and desert mountain subspecies at rest. As such, air temperatures lower than the critical lower critical temperature are expected to elicit cold-induced thermogenesis (e.g. shivering), while warmer temperatures should not elicit a stress response. Animals were acclimated to constant temperature conditions for 24 h prior to experimental measurement.

### Metabolic phenotyping

We measured metabolic variables using an 8-cage FMS manufactured by SSI. Instruments were zeroed and calibrated following the SSI Instrument Settings and Calibration manual. CO_2_-less, dry tank gas was used to calibrate both the O_2_ (to 20.95%) and water vapor (kPa) sensors. The CO_2_ sensor was span calibrated using tank gas of a known concentration (1% CO_2_). Rates of oxygen consumption (*V̇*_O_2__, ml min^−1^) and carbon dioxide production (*V̇*_CO_2__, ml min^−1^) and water vapor levels (mg) were recorded continuously across a 72 h cycle for one empty baseline chamber (e.g. ambient air) and seven animal chambers (each 9.5 l). Instruments were zeroed and spanned between each 72 h experiment. Air was pulled from flow-through chambers at a rate of 1600 ml min^−1^ (96 l h^−1^) by a SSI SS-4 Sub-Sampler Pump (one for each chamber), multiplexed through the SSI MUXSCAN, and sub-sampled at 250 ml min^−1^. A flow rate of 1600 ml min^−1^ and chamber volume of 9.5 l equate to a time constant of 5.90 min to equilibrate 63% of the chamber volume (Lighton, 2017); 95% equilibration therefore takes 16.85 min and 99% takes 25.90 min (Lighton, 2017). Chamber sampling alternated between a baseline chamber measurement for 120 s and a random animal chamber measurement for 120 s each, resulting in approximately two measurements per animal per hour. Frequent baseline measurements were important for reducing measurement noise introduced by environmental transitions. This protocol was repeated twice for each sex under each treatment (diurnal, warm, cool), resulting in 72 h of continuous metabolic measurements for 14 adult males and 14 adult females for each treatment (84 mice total). Barometric pressure (BP, Torr; 1 Torr≈133 Pa), temperature (°C) and RH (%) of the environmental chamber were recorded throughout. Raw data were processed using two macros (available on Dryad) in the ExpeData analytical software (v1.8.4, SSI). The first macro step calculated *V̇*_O_2__ (ml min^−1^), *V̇*_CO_2__ (ml min^−1^) and rate of water loss (RWL; mg h^−1^ at standard temperature and pressure, STP) with smoothing, *z*-transformation (following Lighton, 2008; Lighton and Halsey, 2011; Gerson et al., 2020), lag correction (O_2_/CO_2_, 18 s lag; RWL, 12 s lag) and gas measurements and flow rates adjusted for BP and water vapor (humidity). The second macro step averaged chamber measurements across the most stable 50% of each 120 s measurement window to produce a single average value for each metric within each window. Together, these filtering steps remove excess variation caused by excretion events. Total energy expenditure (TEE) and *V̇*_O_2__ were used as proxies for metabolic rate (MR), as per Lighton (2019) and Hulbert and Else (2004), respectively. As a measure of metabolic fuel use, the respiratory quotient (RQ), or respiratory exchange rate (RER), was calculated as the ratio of *V̇*_CO_2__ to *V̇*_O_2__ (Lighton, 2017). TEE (kcal h^−1^) is the sum of the basal metabolic rate (BMR), physical activity (typically, 4–16%; Abreu-Vieira et al., 2015) and cold-induced thermogenesis, and was calculated as in Lighton (2017, equation 9.15). BMR is difficult to measure in free-living mice because of the confounding effects of activity, constant flux in digestive state and variation in body temperature (Abreu-Vieira et al., 2015), which were not directly measured in this study. Measurements located more than three standard deviations away from the mean for each response variable were identified as outliers, presumed to be non-biological in nature or possibly driven by excretion events, and were removed from downstream analyses.

### Statistical analysis

All analyses were conducted in R v.3.6.1 (http://www.R-project.org/), unless otherwise specified. All code is freely available from GitHub (www.github.com/jpcolella/peer_respo). After first confirming that the data were normally distributed (*shapiro_test* function in the package *rstatix* v.0.6.0; https://CRAN.R-project.org/package=rstatix) and had homogeneous variance (*stats::var.test*), we ran an unpaired two-sample *t*-test (*stats::t.test*, *P*<0.05) to test for sexual dimorphism in mass. Data were subset by sex, experiment and time interval (morning transition, light phase, evening transition, dark phase) to generate summary statistics. We calculated the mean±s.d., median and range (minimum, maximum) for each time interval, experiment and sex. We used Student's two-tailed *t*-test (*t.test*), adjusted for multiple comparisons (Bonferroni correction, *p.adjust*), to test for significant (*P*<0.05) differences in response variables between the sexes under each experimental condition. Significant differences in male and female responses led us to analyze each sex independently in all downstream analyses.

**Fig. 2.**
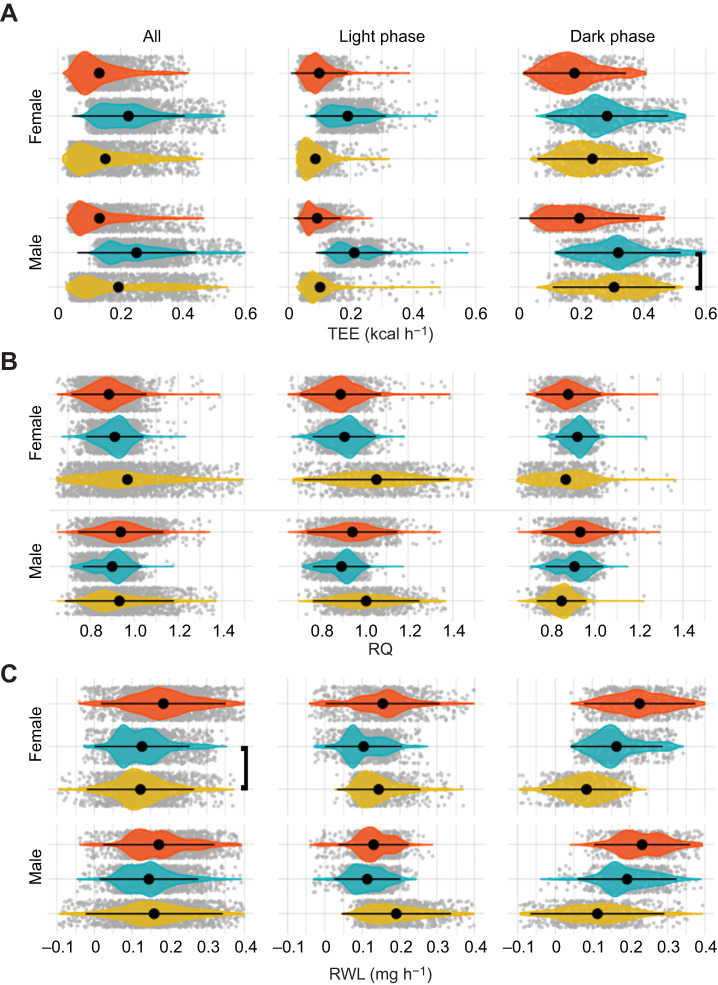
**Differences in metabolic response variables between treatment groups.** Violin plots show mean±s.d. (black dot and horizontal line) total energy expenditure (TEE; A), respiratory quotient (RQ; B) and water loss rate (RWL; C). Individual values are represented by gray dots. All but two group means were significantly different (Bonferroni corrected, Wilcoxon test, *P*<0.05; the insignificant comparisons are bracketed]. Left: entire 24 h cycle (‘All’); center: light phase only; right: dark phase only. Treatment groups: diurnally variable, yellow; constant warm, orange; constant cool, blue.

To determine whether mass was a covariate, we ran a general linear regression between mass and each response variable to test for a significant relationship (*R*^2^, *P*<0.05). Because metabolic response variables varied significantly between the light and dark phases, we further tested for an association between mass and each variable during only the light and dark phases separately. Mass was not a significant predictor of metabolic responses overall in our study and was therefore not included as a covariate in an ANCOVA. We evaluated ANOVA assumptions in R, including: independent observations, no significant outliers, data normality and homogeneity of variance (see Supplementary Materials and Methods for further details; Table S1). Because of assumption violations, we instead ran a Kruskal–Wallis test (*stats::kruskal.test*) as a non-parametric alternative to a one-way ANOVA (Hollander and Wolfe, 1973). The Kruskal–Wallis test extends the two-sample Wilcoxon test to situations where there are more than two groups, to identify significant differences in group means across experimental treatments (*P*<0.05, Bonferroni corrected; Fig. 2).

### Time-series analyses

Change point analyses were used detect significant transitions in the mean and variance (*changepoint::cpt.meanvar*) of each response variable across a 24 h cycle (*changepoint* package v.2.2.2; Killick and Eckley, 2014; https://CRAN.R-project.org/package=changepoint). Change points identified under diurnally variable environmental conditions were compared with those detected under constant warm and cool treatment groups to determine whether organismal responses were anticipatory or reactive relative to environmental change and whether these shifts remained unchanged (or not) across experimental conditions. Additional parameters almost always improve change point model fit; therefore, it is important to quantitatively identify meaningful penalty thresholds to avoid overparameterization (Killick and Eckley, 2014; Haynes et al., 2017). Selection of penalty thresholds depends on many factors, including the size and duration of change, which remain unknown prior to analysis (Guyon and Yao, 1999; Lavielle, 2005; Birgé and Massart, 2007). Current best practices rely on visual inspection of change point estimates compared with time series data and known forcing functions, if available (Killick and Eckley, 2014). For example, there are two known (artificially controlled) environmental transitions under the diurnally variable treatment; therefore, a minimum of two change points, maximum of four (corresponding to the beginning and end of each transition), is expected and more or fewer identified change points should be interpreted with caution and explored in greater detail (Killick and Eckley, 2014). Sharp transitions in slope are especially difficult to fit into discrete bins, and may cause additional change points, that are not biologically relevant, to be estimated within steep environmental transitions (Fearnhead et al., 2019). To estimate penalty thresholds, we used the CROPS (Change points for a Range Of PenaltieS; Haynes et al., 2017) penalty setting with the PELT (Pruned Exact Linear Time; Killick et al., 2012) method to test a range of penalty values (λ, 5–500) and optimize computational efficiency. To balance improved model fit with the addition of more parameters, we plotted the difference in the test statistic versus the number of change points detected to identify an optimal penalty threshold of 20 as appropriate for all but one comparison (e.g. Δlog-likelihood>λ). Diagnostic plots for baseline male water loss did not asymptote with the *x*-axis under a penalty threshold of 20, thus we used a penalty value of 18 for this test. Under the assumption that a large improvement in model fit is indicative of a true change point (Lavielle, 2005; Hocking and Killick, 2017), we used Akaike information criterion (AIC) diagnostic plots to identify the optimal number of change points based on our data (maximum-likelihood). Visual inspection of change points plotted over our raw data allowed us to identify change points that may be overfitted. As a Bayesian alternative, we also used the *bcp* package v.4.0.3 (Erdman and Emerson, 2007) to estimate change points based on a posterior probability threshold of 0.2. The occurrence time of significant change points was compared across treatment groups and against known room transitions to estimate lag-times and identify anticipatory versus reactionary metabolic responses. The mean and slope of each segment were calculated in R.

Time-series data were visualized in *ggplot2* v.3.3.0 (Wickham, 2016) and *visreg* v.2.7.0 (Breheny and Burchett, 2017) across a 24 h period using both local regressions, as a non-parametric approach that fits multiple regressions in a local neighborhood (*loess* option in *ggplot2*), and a generalized additive model (GAM; Wood, 2011) in the *mgcv* package v.1.8-31 [Wood, 2004, 2017; formula: response variable∼s(time), where time is measured in seconds]. Plots were generated in R and edited in the free, open-source vector graphics editor InkScape (https://inkscape.org). Distributions were contrasted across treatment groups.

## RESULTS

### Metabolic phenotyping

In total, we recorded 13,020, 120 s intervals (6550 female, 6470 male). We recorded 4020 observations under diurnally variable environmental conditions (2038 female, 1982 male). We measured metabolic variables for 4506, 120 s intervals under the constant cool treatment (2256 female and 2250 male). For the constant warm treatment, we recorded 4494 total observations (2256 female, 2238 male). After removing outliers, we retained 6335 female measurements (1877 measurements under diurnally variable environmental conditions, 2230 under constant warm conditions, and 2228 under constant cool conditions). We retained 6255 measurements for males (1844 diurnally variable, 2214 constant warm and 2197 constant cool). Female mass ranged from 15.4 to 25.2 g and averaged 21.8, 21.7 and 20.0 g for warm, cool and diurnally variable experiments, respectively. Male mass ranged from 15.5 to 26.6 g, averaging 22.0, 21.0 and 22.2 g across the three experiments. All raw data (Expedata files) and processed machine-readable csv files are available from Dryad (https://doi.org/10.5061/dryad.f4qrfj6v0).

### Static statistics

No significant difference was found between mean mass for males and females, and there was no relationship between mass and any measured metabolic variable overall or within light and dark cycles, as expected given the narrow mass range. *t*-tests between the sexes identified significant differences in metabolic response variables, with few exceptions (Fig. 2; Fig. S1). Summary statistics for each metabolic response variable for each sex and experimental group (diurnal, constant warm, constant cool) are reported in Table 1 (TEE, RQ, RWL) and Table S2 (*V̇*_O_2__, *V̇*_CO_2__). Based on mass, our results are consistent with White and Seymour's (2003) TEE predictions (∼0.17 kcal h^−1^ or 0.2 W). Mean TEE was higher for males than for females under all experimental conditions, while mean RWL was only higher for males under the constant cool treatment. The RQ was roughly equivalent to the FQ across all experimental treatments, but patterning (e.g. the direction, amplitude and relative timing of distributional change) differed. Under diurnal conditions, RQ patterning was similar between the sexes. Both males and females reached an RQ greater than 1 during the light period, while RQ dropped below the FQ during the dark period (Table 1). Under both constant temperature treatments, RQ was stable, just above the FQ under constant cool conditions and equal to (females) or just above (males) the FQ under constant warm conditions (Table 1).

**Table 1.**
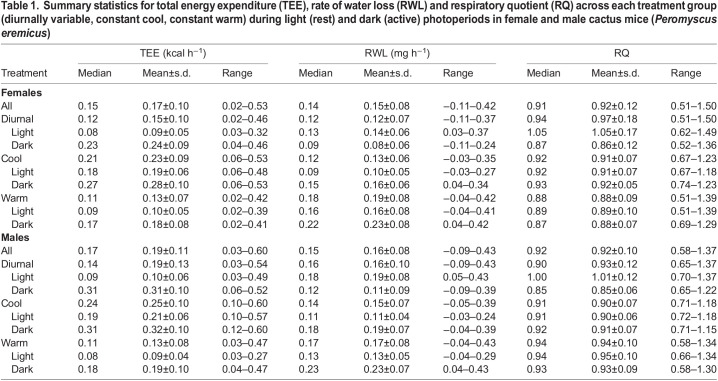
**Summary statistics for total energy expenditure (TEE), rate of water loss (RWL) and respiratory quotient (RQ) across each treatment group (diurnally variable, constant cool, constant warm) during light (rest) and dark (active) photoperiods in female and male cactus mice (*Peromyscus eremicus*)**

Within each sex, the majority of pairwise tests comparing metabolic variables between treatment groups were significantly different (Kruskal–Wallis, Fig. 2; *t*-test, Fig. S1). Separating each treatment into light and dark intervals also revealed evidence of significantly different physiological responses among treatment groups, with few exceptions (Fig. 2; Fig. S1). Results were visualized as violin plots to illustrate variance and group means across each variable and treatment group (Fig. 2). During the light period, diurnally variable conditions were equivalent to those of the constant warm treatment, yet metabolic responses differed for TEE, RQ and RWL (Table 1). Male TEE and *V̇*_O_2__ did not differ between dark phase diurnally variable conditions and the constant cool treatment, during which the environmental conditions were identical, but these two time intervals surprisingly differed significantly in all other metabolic variables examined (not shown). Mean TEE was higher during the dark period, relative to light, for all experiments. Both sexes exhibited their highest mean TEE under constant cool conditions (females 0.23 kcal h^−1^, males 0.25 kcal h^−1^) and lowest under constant warm conditions (both 0.13 kcal h^−1^). Females lost significantly less water under diurnally variable environmental conditions (mean 0.12 mg h^−1^) than they did under constant warm (0.19 mg h^−1^) or cool (0.13 mg h^−1^) treatments, while males lost significantly less water under the constant cool treatment (0.15 mg h^−1^), relative to diurnally variable (0.16 mg h^−1^) or warm (0.17 mg h^−1^) treatments. Female water loss did not differ significantly between diurnally variable and cool treatment groups; however, differences were detected when the data were split into light and dark periods. Greatest water loss was observed under the constant warm treatment; however, peak RWL (0.43 mg h^−1^ for males, 0.37 mg h^−1^ for females) occurred under diurnally variable conditions.

### Signal processing of physiological time-series data

**Fig. 3.**
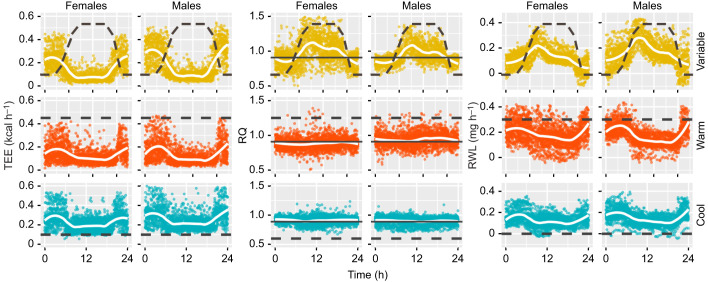
**Metabolic responses across a 24** **h cycle for males and females in the three treatment groups.** Treatment groups: diurnally variable, yellow; constant warm, orange; constant cool, blue. Black dashed lines illustrate daily temperature variation across experiments (detailed in Fig. 1) and solid lines (RQ plots only) indicate the food quotient (FQ=0.89). Individual values are represented by colored dots, and white lines are a smoothed, locally weighted regression (LOESS).

Time-series data for TEE, RQ and RWL are shown in Fig. 3 for all experiments and both sexes across a 24 h cycle (*V̇*_O_2__ and *V̇*_CO_2__, Fig. S2). Both sexes exhibited similar physiological patterning of TEE across all treatment groups, but at different magnitudes of change (Fig. 3, Table 1). RQ was highly variable under diurnal environmental cycling, but flat-lined at the FQ (0.89) under both constant conditions (Fig. 3). An RQ of 1.0 indicates carbohydrate catabolism, whereas an RQ of 0.8 indicates either increased protein catabolism or a mixture of lipid and carbohydrate catabolism, and pure lipid catabolism produces an RQ of 0.7 (Richardson, 1929). Under constant temperatures, RQ was stable at 0.91±0.07 for females and 0.90±0.07 for males under constant warm conditions, and 0.88±0.07 and 0.94±0.10 for females and males, respectively, under constant cool conditions. Only under diurnal environmental cycling did RQ reach values above 1.0, averaging 1.00 for males and 1.05 for females during the light period and reaching a peak of 1.37 and 1.50 for males and females, respectively. Patterns of RWL were inverted between diurnally variable and constant treatments (Fig. 3). Under diurnally variable conditions, RWL increased in the morning, decreased throughout the warm light phase and stabilized during the cool dark period. In contrast, under constant temperature and humidity, water loss mirrored TEE patterning, decreasing during the light period and increasing during the dark period when animals resumed higher levels of activity. Similar patterns were also observed in *V̇*_O_2__ and *V̇*_CO_2__.

**Fig. 4.**
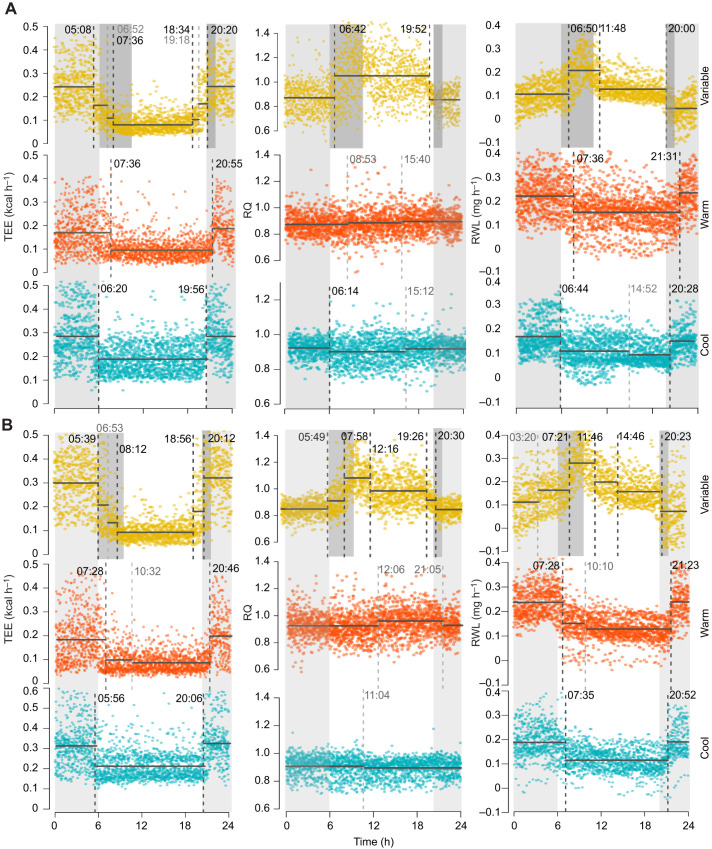
**Change point estimates for metabolic responses across a 24 h cycle for males and females in the three treatment groups.** Change point estimates (vertical dashed bars; h:min) denote shifts in mean and variance for TEE, RQ and RWL for females (A) and males (B) in each treatment group (diurnally variable, yellow; constant warm, orange; constant cool, blue). Light gray dashed vertical lines indicate potentially overfitted change points. Light gray shading indicates the dark phase, dark gray shading indicates transition periods (present only under diurnally variable conditions), no shading indicates the light phase. Individual values are represented by colored dots.

AIC-optimized, maximum-likelihood change point estimates are given in Table 2 and presented in Fig. 4 (*V̇*_O_2__ and *V̇*_CO_2__, Fig. S2). Under diurnal environmental cycling, males exhibited an average of five change points and females exhibited three. As expected when fitting continuous data to a stepwise model, both sexes had change points overfitted within environmental transition periods (Fig. 4). Metabolic change points roughly correspond to the initiation and conclusion of physical environmental transitions (morning, evening). Some additional change points were detected outside of environmental transition periods, including a large downshift in RWL present in both sexes during the peak of the light period under diurnally variable conditions. On average, females exhibited a morning shift in TEE, *V̇*_CO_2__ and *V̇*_O_2__ earlier (05:16 h) than males (05:41 h). Under diurnally variable conditions, morning change points for both sexes occurred prior to physical changes in temperature and humidity within the environment. Females experienced a second metabolic shift around 07:00 h and males just after 08:00 h. Evening shifts also occurred in two stages: females experience an uptick in TEE, *V̇*_CO_2__ and *V̇*_O_2__ just after 18:30 h, and males just before 19:00 h, followed by a secondary shift around 20:12 h for both sexes. We observed a single, large upward morning shift in RWL during the morning environmental transition. The initial uptick was followed by at least one downshift in RWL during the light period, followed by an additional downshift coincident with (20:00 h females) or soon after (20:23 h males) the initiation of the evening transition. Females exhibited only two change points in RQ under diurnally variable conditions: one during the morning transition, after the change in photoperiod and environment, and another preemptive of the evening transition. Males exhibited five AIC-optimal change points in RQ under diurnally variable conditions: one just before and one during each transition (morning, evening) and another in the middle of the light phase coincident with a downshift in RWL. Means and standard deviations for each segment (i.e. period of mean and variance stability between estimated change points) are reported in Table S3 both for the optimal number of change points and excluding potentially overfitted change points. Bayesian change point results (Tables S3 and S4) are generally concordant with maximum-likelihood estimates. Decreasing the time constant, either by reducing chamber size or increasing flow rates, will increase the accuracy of these time estimates by ensuring complete turnover of chamber air volume and equilibration.

**Table 2.**
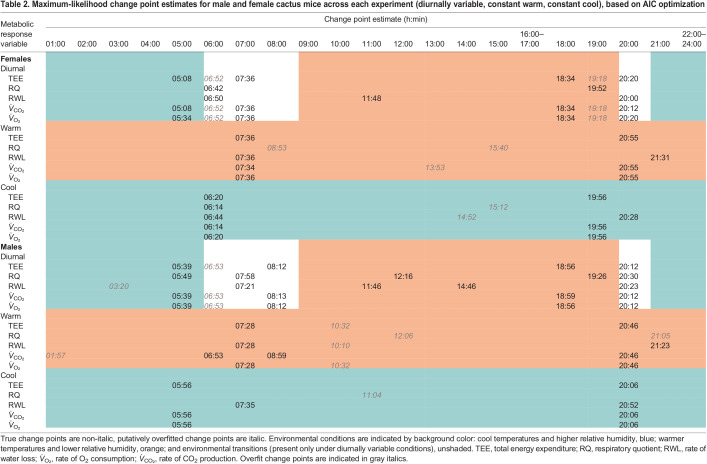
Maximum-likelihood change point estimates for male and female cactus mice across each experiment (diurnally variable, constant warm, constant cool), based on AIC optimization

Under constant warm conditions, both sexes experienced delayed (>2 h on average) metabolic shifts relative to those recorded under diurnally variable conditions and all major shifts occurred after changes in photoperiod. Under constant cool conditions, however, the morning transition occurred later than it had under diurnal conditions for both sexes, but the shift remained preemptive of photoperiod change in males. Evening metabolic transitions under constant cool conditions also moved forward in time, but under this condition, the female shift occurred prior to a change in photoperiod, while the male shift occurred after.

## DISCUSSION

In this study, we compared metabolic responses of male and female cactus mice under diurnally variable and constant environmental conditions to assess the influence of temperature and humidity on adaptive metabolic regulation, when photoperiod and access resources remain constant. Overall, metabolic patterning was significantly shaped by environmental conditions, females were less prone to dehydration relative to males, and metabolic responses were best optimized under circadian conditions that mimic the species’ native environment. An RQ greater than 1.0, suggestive of lipogenesis, was observed only during the light phase under circadian environmental conditions, supporting the hypothesis that *P. eremicus* exhibits circadian torpor, but suggesting it is not mediated by resource restriction as was previously hypothesized (MacMillen, 1965; Morhardt and Hudson, 1966). Many of the differences recorded herein would not have been observed under constant environmental conditions, as metabolism is traditionally measured, and therefore highlight the importance of continuous metabolic phenotyping through environmental transitions to better understand the physiological responses of organisms to dynamic environments.

### Metabolic sexual dimorphism

Previous studies have found different results for sexual dimorphism in this species (females slightly outweigh males: Davis, 1966; no dimorphism: Manning et al., 2006). We did not detect body mass dimorphism between the sexes, nor a correlation between mass and any measured metabolic variable. Despite the lack of size dimorphism, metabolic responses varied significantly between non-reproductive adult males and females (Fig. S1). Although less expected in the absence of size differences, metabolic demands frequently vary between sexes (Mittendorfer, 2005; McPherson and Chenoweth, 2012; Carmona-Alcocer et al., 2012), which may be explained by differences in behavior, activity level or body composition (McDonald et al., 1989; Chappell et al., 2004). Body temperature, not measured as part of this study, is maintained relatively independent of ambient temperature (McNab and Morrison, 1963). At low temperatures, *Peromyscus* can substitute exercise-generated heat for thermogenic heat by scaling activity levels, but this only leads to small energetic savings (Chappell et al., 2004). Sex-specific differences in body composition, not measured as part of this study, may also lead to differential heat conservation between the sexes (McDonald et al., 1989) and this remains to be explored in detail for this species. In general, females spent less energy than males (Table 1, Fig. 2), also consuming more O_2_, producing more CO_2_ and losing more water than males. Nonetheless, the overall pattern of variation, including the direction, amplitude and frequency of change across all metabolic response variables, was similar among the sexes for each treatment group (Fig. 3).

Under diurnally variable environmental conditions, RWL varied most between the sexes: males lost significantly more water than females. This observation may be in part explained by higher activity levels in males (observed but not quantitatively recorded), as increased activity levels lead to greater evaporative water loss through increased respiration (Bruck, 1962). Elevated water loss may allow males to dissipate heat more quickly through evaporative cooling (e.g. panting, saliva spreading), but could also lead to reduced male survival as a result of dehydration following rapid loss of body water. Under constant warm conditions, females lost more water than males during the light phase despite similar levels of TEE and conditions identical to those of the diurnally variable light phase (Table 1). This suggests that females may be less tolerant of extended heat stress. The opposite pattern was observed for males, which appear to be less cold tolerant. Relative to females, males spent significantly more energy and lost more water under constant cool conditions (Table 1). This relationship may be a consequence of elevated thermogenesis in males or perhaps females are able to sustain lower body temperatures, potentially as a consequence of sex-specific differences in body composition (McDonald et al., 1989; Beaudry and McClelland, 2010; Gordon, 2012). In mammals, males generally have a lower proportion of body fat relative to females, and therefore rely more on active thermogenesis to maintain core body temperature at cooler ambient temperatures. Thermogenesis in *Peromyscus* relies heavily on fats (Cheviron et al., 2012; Munshi-South and Richardson, 2017), the catabolism of which reduces RQ values as a result of increased O_2_ investment in fat oxidation. Consistent with increased fat catabolism for thermogenesis, males exhibited lower RQ values under constant cool conditions, relative to females (Table 1). Even though only non-reproductive individuals were examined in this study, differences in reproductive biology or sex-specific selection may have shaped metabolic evolution. Sexual dimorphism in heat and dehydration tolerance is consistent with observations in other mammals, including humans (Glucksmann, 1974; Mittendorfer, 2005; Pomatto et al., 2018), but counters early observations of *P. eremicus* by McNab and Morrison (1963) where males expired under upper critical temperatures, while females survived. In males, a body temperature increase of only a few degrees centigrade above normothermia can reduce the viability of stored spermatozoa (Moore, 1926), a challenge typically resolved by externalizing the testes to the scrotum. Even in cases of extreme heat, hyperthermia usually results in only temporary male infertility, as spermatozoa are quickly replaced; in contrast, the fitness consequences of hyperthermia are greater for females, where the embryo cannot be thermally isolated and damage is irreversible (Greenwood and Wheeler, 1985). In consequence, sex-specific selection may lead to differences in traits that do not initially appear to be directly associated with reproductive function, such as growth rate, thermoregulation, metabolic biorhythms and environmental sensing (Glucksmann, 1974; McPherson and Chenoweth, 2012; Juárez-Tapia and Miranda-Anaya, 2017; Calisi et al., 2018). Differences in lipid kinetics are hypothesized to maintain sexually dimorphic metabolic phenotypes (Mittendorfer, 2005) and genes involved in lipid metabolism have been identified as important for thermoregulation in high-altitude adapted *Peromyscus* (Cheviron et al., 2012). A similar suite of genes was also identified as under selection in wild cactus mice (Tigano et al., 2020) and related canyon mice (Colella et al., 2021a), reinforcing a potentially important role for sex-specific lipid metabolism in the physiological adaptation of this species to hot and dry environments.

### Circadian metabolic tuning

We hypothesized that desert-adapted cactus mice would exhibit physiological and behavioral responses that minimize water loss and limit TEE under warm, arid conditions (MacMillen, 1965, 1983). Indeed, we found significant support for environmentally associated adaptive metabolic responses for all examined variables. Circadian patterning of TEE was observed across all experimental treatments, consistent with the nocturnal activity pattern of this species (Warner et al., 2010; Refinetti, 2010): higher dark phase TEE, lower light phase TEE. In mammals, activity patterns, like nocturnality, shape patterns of circadian metabolism and are frequently governed by photoperiod (Miranda-Anaya et al., 2019), the role of which remains to be explored for this species. In deserts, increased locomotion and foraging during the dark phase minimizes evaporative water loss during the warmer, drier light phase when physiological cooling is most challenging (Abreu-Vieira et al., 2015). Mammals, including near relatives of *Peromyscus*, may also maintain warmer body temperatures during active periods, relative to inactive periods, which cannot be fully explained by differences in activity levels, but may instead require additional metabolic resources to achieve (Abreu-Vieira et al., 2015). TEE is the sum of an organism's BMR, physical activity and energy invested in cold-induced thermogenesis (e.g. shivering). In this context, TEE represents only a coarse approximation for differences in activity level. At thermoneutrality, mice (*Mus*) expend approximately 60% of their TEE on BMR, 12% on the thermic effects of food, 25% on physical activity and 0% on cold-induced thermogenesis (Abreu-Vieira et al., 2015). Heat-dependent torpor or cold-induced thermogenesis (Chappell et al., 2004; Storz et al., 2019; Bastías-Pérez et al., 2020) likely explain elevated TEE observed under constant cool conditions, as well as the greater variance in TEE observed under diurnally variable environmental conditions. High summer surface temperatures are a hypothesized cue for daily torpor initiation in cactus mice (MacMillen, 1983) and thermogenesis is expected to occur during the dark phase when temperatures near the lower critical temperature threshold for this species (Table 1).

In a captive environment where food is not a limiting resource, cactus mice spent less energy during the dark phase under constant warm conditions than they did under diurnally variable conditions, which suggests that extreme or extended temperature events may shape cactus mouse foraging strategies or activity patterns in the future (Du Plessis et al., 2012; Mason et al., 2017; Riddell et al., 2019, 2021). Behavioral thermoregulation can be achieved through increasing or decreasing activity or by relocating to a thermally suitable microclimate (e.g. underground burrow) to buffer against excessive heat (Riddell et al., 2021). While behavioral thermoregulation is energetically less expensive compared with autonomic thermoregulation (Terrien et al., 2011; Hayford et al., 2015), there is a tradeoff between the energy and time invested in thermoregulation and that which remains available for other biological processes critical to survival. Increased investment in thermoregulation diverts time and energy away from reproduction and resource acquisition, and physical relocation to a suitable microclimate costs energy and may drive animals further away from areas that are favorable in terms of resource availability or predation risk (Dunbar, 1998; Kearney and Porter, 2004; Mason et al., 2017).

Under diurnally variable environmental conditions, both sexes experienced greater variance in all metabolic response variables compared with constant conditions, indicating that metabolic regulation is both dynamic and environmentally mediated. Cactus mice more efficiently partition energy, water and fuel under diurnally variable environmental conditions, relative to artificially constant conditions. For example, dark phase RWL and TEE were reduced under diurnally variable conditions relative to constant temperature treatments, despite consistently high dark phase TEE across all treatments. As expected, RWL was greatest under constant warm, arid conditions and lowest under constant cool, more humid conditions (Table 1). When split into light and dark periods, animals in the diurnally variable treatment group lost significantly less water (Table 1), even when comparing between equivalent environmental conditions (e.g. diurnally variable light phase versus constant warm conditions, diurnally variable dark phase versus constant cool conditions). In fact, during the warm, dry light phase of the diurnally variable treatment, animals lost even less water than they did under constant cool conditions, suggesting the water economy of cactus mice is effectively tuned to natural diurnal cycling of temperature and humidity, or when the animals do not experience extended environmental stress. Although significant, the difference in RWL between treatments is small (0.01–0.07 mg h^−1^), and the biological consequences of these differences remain to be experimentally tested.

### Timing of metabolic shifts

Analysis of continuous metabolic phenotypes confirmed that cactus mouse physiology is well tuned to circadian patterns of environmental variation. We expected to detect a physiological lag between the sensation of environmental change and a measurable metabolic response (Aguilar-Roblero, 2015). For example, in a related cricetid rodent, *Neotomodon alstoni*, increased activity levels were observed immediately following the initiation of the dark phase (Miranda-Anaya et al., 2019). In contrast, under normal circadian environmental cycling, most metabolic shifts in cactus mice occurred in advance of physical environmental change, or the perception thereof (Fig. 3; Fig. S2). Evidence of anticipatory metabolic shifts suggests that biorhythm entrainment is adaptive, particularly in highly variable environments such as deserts.

The timing of most metabolic shifts was disrupted under constant temperature conditions; thus, circadian torpor is not exclusively regulated by access to food, ambient temperature or relative humidity. The persistence of two major metabolic shifts under both constant treatments, however, suggests that photoperiod may be at least in part responsible for maintaining metabolic biorhythms. Photic synchronization is common among mammals (Miranda-Anaya et al., 2019). Observed temporal changes in metabolism between diurnally variable and constant treatment groups were also consistent with males being more prone to dehydration than females. Relative to males, females used less energy and lost less water under cool conditions, presumably as a result of less investment in active thermogenesis, but females experienced a longer period of dark phase activity under constant warm conditions, which may be maladaptive. Experiments with inverted light and dark phases will be necessary to test the strength of the observed circadian rhythm and role of photoperiod.

### Lipogenesis as a potential buffer against dehydration

Under diurnally variable conditions, RQ increased with increasing temperature and humidity, and then downshifted in the evening, coincident with or just prior to the evening transition to cooler, more humid conditions (Figs 3 and 4). In contrast, RQ remained constant, at the FQ, under both constant temperature–humidity conditions. This indicates that RQ in cactus mice is tightly controlled by ambient temperature and, more specifically, sustained temperature.

An RQ greater than 1.0 can indicate either activity levels above aerobic threshold (Whipp, 2007; Zagatto et al., 2012) or lipogenesis (Benedict and Lee, 1937; Kleiber, 1961; Elia and Livesey, 1988; Abreu-Vieira et al., 2015; Levin et al., 2017). For cactus mice, elevated RQ levels were observed only during the light phase, when mice are inactive, a pattern suggestive of facultative lipogenesis. Glycolysis converts carbohydrates (glucose) into pyruvate, which is then converted into acetyl-CoA. Excess acetyl-CoA can be used to produce fatty acids and triglycerides through the process of lipogenesis. During this process, glycerol hydroxyl groups (-OH) react with the carboxyl end (-COOH) of a fatty acid chain to produce water (H_2_O) and carbon (C) atoms that bind to oxygen (O) through a dehydration synthesis reaction, resulting in the formation of water and carbon dioxide (Ameer et al., 2014; OpenStax, 2016), which leads to a spike in RQ values. As free water is scarce in desert environments, we hypothesized that lipogenesis may be an important indirect source of water for cactus mice and other desert taxa (Bradley and Yousef, 1972). Endogenous, circadian synthesis of fatty acids and triglycerides may buffer against the negative physiological effects of dehydration, which are expected to be most intense during the warm, but inactive light phase. If circadian lipogenesis was driven by an energy imbalance, dark phase refeeding followed by light phase inactivity and fasting, we would expect similarly high RQ values under both constant temperature treatments. Under this scenario, RQ values would be highest for the constant warm treatment where animals rely less on thermogenic fat catabolism for thermogenesis. Yet, interestingly, an RQ greater than 1.0, consistent with lipogenesis, was only observed during the light phase under diurnally variable environmental conditions and surprisingly, never during either constant temperature treatment. Circadian cycling of lipogenesis has been observed in other rodents (hamsters, rats, mice), modulated by dietary and hormonal variation (insulin and prolactin: Kimura et al., 1970; Cincotta and Meier, 1985; Bryson et al., 1993; Kersten, 2001; Letexier et al., 2003; Abreu-Vieira et al., 2015). Insulin-associated genes have also been shown to be under selection in cactus mice (*Insl3*, *Igfbp3*: Kordonowy et al., 2017) and *Inpp5k* appears to be a functionally important transcript for water regulation in this species, also involved in insulin signaling (MacManes, 2017; Bridges and Saltiel, 2015).

Circadian patterning of RQ under environmentally variable conditions (elevated during the inactive, warm light phase; depressed during the active, cool dark phase) is suggestive of circadian torpor, as described by Macmillen (1965, 1972). While the abiotic regulatory mechanism(s) of lipogenesis in cactus mice remains elusive, it is not driven by photoperiod, nor constant temperature or humidity. Circadian lipogenesis is also not resource dependent, as originally hypothesized (Macmillen, 1965, 1972; see also Stephan, 2001), because animals were provided with food and water *ad libitum* throughout the experiments. Lipogenesis is controlled by hepatic insulin levels (Ameer et al., 2014), and insulin levels are expected to become depleted over the course of 24 h, with dark phase refeeding increasing serum insulin again and, in turn, potentially re-stimulating lipogenesis (Kimura et al., 1970; Bryson et al., 1993; Yamajuku et al., 2012; Liu et al., 2013; Adamovich et al., 2014). However, the absence of an RQ greater than 1.0 under either constant treatment suggests that circadian torpor is not solely mediated by insulin availability but also depends to some degree on environmental periodicity. Extended exposure to sub-optimal environmental conditions can negatively impact body condition and survival in endotherms (McKechnie and Wolf, 2010; Gardner et al., 2016); therefore, we hypothesize that extended thermal stress may trigger a shift in metabolic resource usage (Taborsky et al., 2021). Environmental stability, in addition to the physiological limits of an organism, is a key factor in shaping stress responses (Taborsky et al., 2021). Continuous metabolic phenotyping through state transitions (multiple days of circadian cycling followed by multiple days of constant conditions) will be necessary to parse environmental factors responsible for circadian torpor.

High altitude-adapted *Peromyscus* exhibit metabolic differences that enhance survival in cold hypoxic environments (Ivy and Scott, 2018, 2021). In these species, cold-induced thermogenesis is used to maintain body temperature and is regulated by flexibility in lipid metabolism (Cheviron et al., 2012; Munshi-South and Richardson, 2017). Lipids are known to regulate circadian rhythmicity in mammals, with hepatic lipid metabolism being controlled by a suite of Clock genes (Eckel-Mahan et al., 2012; Adamovich et al., 2014, 2015), some of which have been identified as under selection in other desert-adapted *Peromyscus* species (*BMAL1*, *BMAL2*: Colella et al., 2021a). While elevated RQ values are highly suggestive of light phase lipogenesis, increased protein catabolism could also elevate RQ over the FQ, to a degree, and contributions of protein remain to be more thoroughly explored as a mechanism of dehydration tolerance in small mammals (e.g. protein-for-water hypothesis: McCue et al., 2017). Protein catabolism is an important water conservation strategy in migratory birds (Gerson and Guglielmo, 2011), and genes involved in protein catabolism have been identified in another desert-adapted *Peromyscus* (*P. crinitus*: Colella et al., 2021a). Year-round high levels of urine concentration in the near-relative *P. crinitus* suggests that canyon mice may subsist on a high-protein diet, which may buffer against the effects of heat and dehydration (MacMillen, 1983). Comparative analyses of urea nitrogen concentrations and diets across *Peromyscus* may prove useful in identifying physiological and behavioral strategies for accommodating high-heat, low-water conditions. Developmental exposure to thermal extremes also shapes adaptive responses (Ivy and Scott, 2021) and remains to be explored for this species.

### Conclusions

In conclusion, our results highlight an important role for circadian environmental variation in the evolution of metabolic efficiency in the desert-adapted cactus mouse. Lipid metabolism is increasingly emerging as a common mechanism of circadian and thermoregulatory plasticity (Rocha et al., 2021), that warrants further experimental investigation in this species through both dietary and environmental manipulation and *in vitro* tests of metabolic fuel usage (Adamovich et al., 2014). Studies of physiology in non-traditional model organisms (Bedford and Hoekstra, 2015) provide a comparative framework for identifying shared physiological responses and mechanisms across mammals. Further, linking molecular mechanisms to physiological responses will afford better characterization of the roles of genetics, the environment and behavior in shaping complex physiological phenotypes (Partch et al., 2014).

## Supplementary Material

Supplementary information
