# Supplementary material for: Disentangling environmental drivers of circadian metabolism in desert-adapted mice
Source: J Exp Biol. 2021 Sep 27;224(18):jeb242529. doi: 10.1242/jeb.242529 (PMC8502254; doi:10.1242/jeb.242529)
Supplement: Supplementary information [file jexbio-224-242529-s1.pdf]

Supplementary Materials and Methods

Although observations under diurnally variable environmental conditions are independent, the same animals were recorded under both constant warm and constant cool conditions, making these observations non-independent and violating ANOVA assumptions. Within each response variable, we performed a secondary screen for outliers (> 3 sd from the mean) and produced another data subset excluding additional outliers. We used a Shapiro-Wilk's test (*shapiro_test* in the *rstatix* package v. 0.6.0; Kassambara 2020) for normality ($p < 0.05$, Bonferroni corrected) for each response variable, raw and log transformed. Levene's test (*rstatix::levene_test*) was used to assess homogeneity of variance, again p-values were Bonferroni corrected for multiple comparisons ($p < 0.05$). Exploratory ANOVA tests (*rstatix::anova_test*) were run for each response variable, using Tukey's Honest Significant Differences (HSD; *rstatix::tukey_hsd*) correction for multiple comparisons ($p < 0.05$).

Table S1. ANOVA pairwise mean differences between treatment groups (variable [var], constant warm, constant cool) based on log-transformed data for each response variable (Total energy expenditure [TEE; kcal h^{-1}], respiratory quotient [RQ], relative water loss [RWL; mg h^{-1}], VO_2 [ml min^{-1}], VCO_2 [ml min^{-1}]) across the entire experiment (All) and light and dark intervals separately. All values are significant ($p < 0.05$) except those highlighted in grey or **bolded** which were not significant for log-transformed or untransformed datasets, respectively. All significance values are corrected for multiple comparisons (Tukey's HSD).

		TEE			RQ			RWL			VO_2			VCO_2		
Sex	Subset	var-cool	var-warm	cool-warm	var-cool	var-warm	cool-warm	var-cool	var-warm	cool-warm	var-cool	var-warm	cool-warm	var-cool	var-warm	cool-warm
Females	All	0.53	-0.07	-0.60	**-0.04**	-0.08	-0.03	0.00	0.05	0.05	0.55	-0.05	-0.59	0.5	-0.12	-0.63
	Light	0.85	0.16	-0.69	-0.14	-0.16	-0.02	-0.04	0.01	0.05	0.88	0.19	-0.69	0.74	**0.03**	-0.71
	Dark	0.2	-0.32	-0.51	0.07	0.03	-0.05	0.07	0.12	0.05	0.18	-0.32	-0.5	0.26	-0.30	-0.55
Males	All	0.42	-0.32	-0.74	-0.03	0.01	0.04	-0.01	**0.01**	0.02	0.43	-0.32	-0.75	0.40	-0.31	-0.71
	Light	0.80	-0.07	-0.87	-0.11	-0.06	0.05	-0.06	-0.05	0.02	0.82	-0.06	-0.88	0.71	-0.12	-0.83
	Dark	0.06	-0.52	-0.58	0.07	0.09	0.02	0.07	0.11	0.03	**0.04**	-0.54	-0.58	0.11	-0.45	-0.56

Table S2. Summary statistics (median = med, mean, standard deviation = sd, range) for females (top) and males (bottom) for carbon dioxide produced (VCO_2 ml min^{-1}) and oxygen consumed (VO_2 ml min^{-1}) across each experiment (diurnal, cool, warm) during the light (rest) and dark (active) photoperiods.

		VCO_2				VO_2			
	Data	med	mean	sd	range	med	mean	sd	range
Females	All	0.45	0.52	0.29	0.06-1.59	0.51	0.58	0.33	0.05-1.82
	**Diurnal**	0.38	0.46	0.28	.07-1.59	0.41	0.51	0.34	0.05-1.59
	Light	0.26	0.29	0.15	0.09-1.12	0.24	0.28	0.16	0.08-1.05
	Dark	0.64	0.69	0.26	0.10-1.39	0.78	0.81	0.30	0.14-1.59
	**Cool**	0.65	0.69	0.28	0.17-1.59	0.71	0.76	0.30	0.19-1.82
	Light	0.55	0.58	0.20	0.17-1.51	0.62	0.64	0.21	0.19-1.59
	Dark	0.82	0.87	0.30	0.17-1.59	0.89	0.95	0.33	0.19-1.82
	**Warm**	0.33	0.39	0.22	0.06-1.24	0.38	0.45	0.25	0.06-1.44
	Light	0.26	0.30	0.14	0.08-1.13	0.31	0.33	0.16	0.08-1.32
	Dark	0.49	0.53	0.24	0.06-1.24	0.57	0.61	0.28	0.06-1.38
Males	All	0.51	0.59	0.34	0.09-1.82	0.57	0.65	0.39	0.09-2.03
	**Diurnal**	0.43	0.58	0.36	0.10-1.62	0.48	0.66	0.43	0.09-1.85
	Light	0.29	0.33	0.17	0.10-1.54	0.29	0.34	0.19	0.09-1.62
	Dark	0.90	0.89	0.30	0.10-1.57	1.07	1.04	0.33	0.20-1.79
	**Cool**	0.72	0.76	0.29	0.34-1.82	0.80	0.85	0.32	0.33-2.03
	Light	0.59	0.64	0.18	0.34-1.71	0.66	0.71	0.21	0.33-1.95
	Dark	0.95	0.97	0.30	0.36-1.82	1.05	1.08	0.34	0.39-2.03
	**Warm**	0.34	0.41	0.26	0.09-1.42	0.36	0.45	0.28	0.10-1.58
	Light	0.27	0.29	0.12	0.10-0.80	0.28	0.31	0.13	0.10-0.90
	Dark	0.56	0.60	0.30	0.13-1.42	0.61	0.65	0.32	0.12-1.58

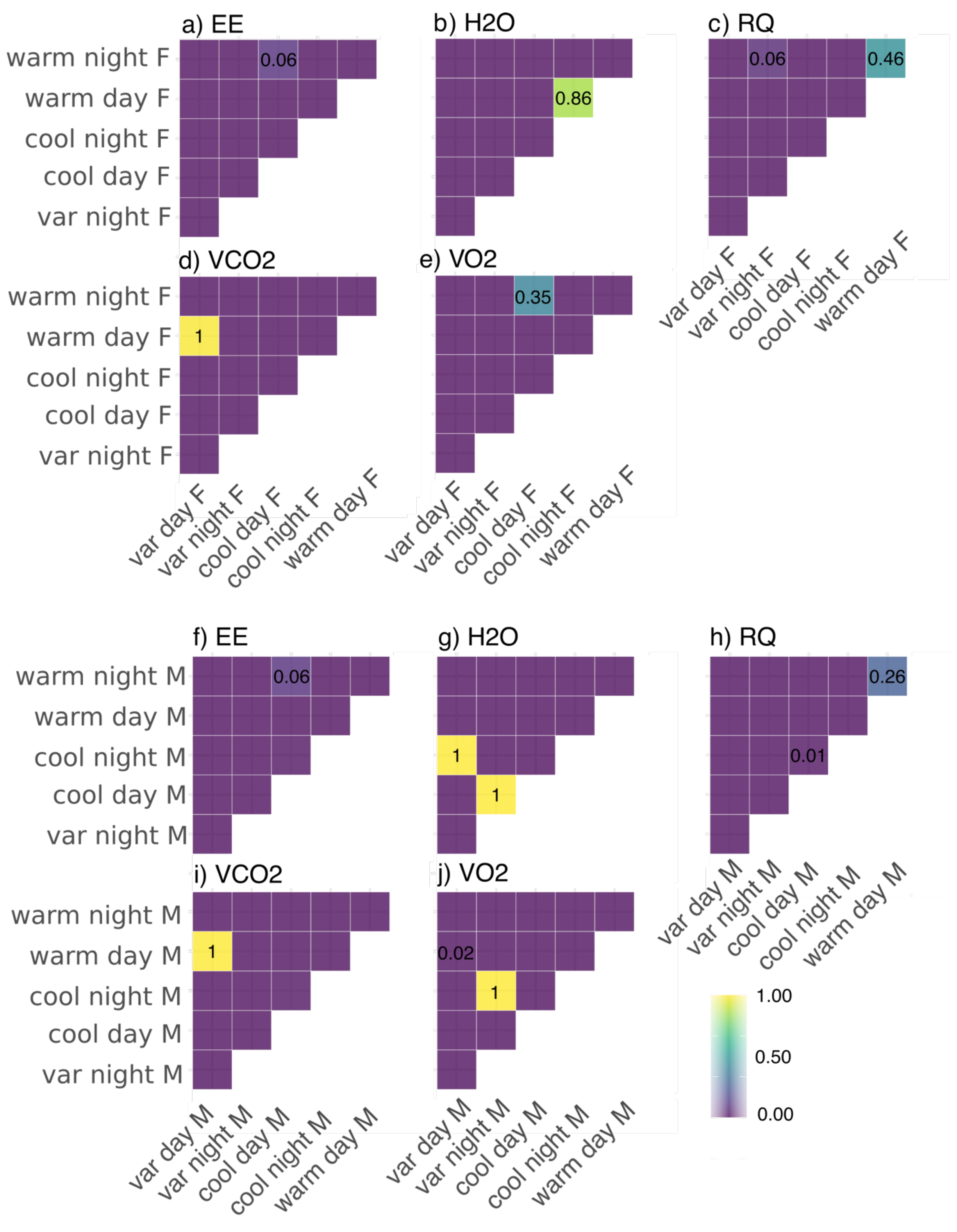

Fig. S1. Heat map of Bonferroni adjusted p-values for pairwise comparisons between dark and light phases f or each experimental group (variable [var], constant warm, constant cool) within each sex (females [F]: a-e; males [M]: f-j), and each response variable (EE, H_2O [RWL], RQ, VCO_2, VO_2).

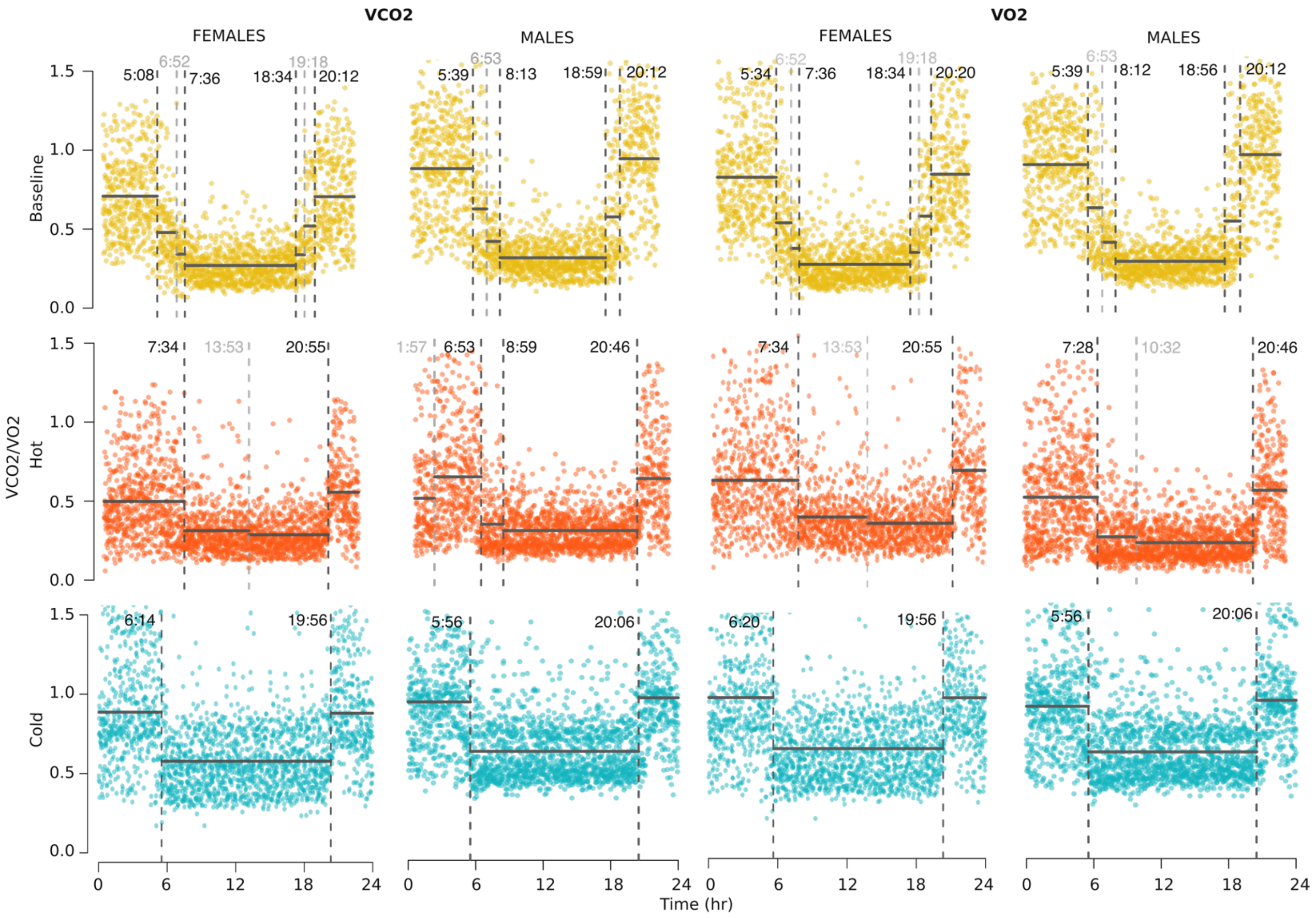

Fig. S2. Change point (hr:min) plots, denoting shifts in mean and variance, estimates for VCO_2 (left) and VO_2 (right) for females and males across a 24 hour cycle. Responses to diurnally variable environmental conditions are in yellow, war conditions in orange, and cool in blue. Vertical, dashed bars indicate change point occurrence, light grey dashed lines indicate potentially over fit change points (Table 2).

Journal of Experimental Biology • Supplementary information

Table S3. Mean of each change point segment (Seg#) for each dependent variable (DV), experiment (Variable, Warm, Cool), and sex (Females, Males). Recalculated segment means, excluding over fit change point estimates (e.g., those shown in grey in Table 2), are shown in the Alt mean column with an X indicating ignored change points.

Exp.	Variable						Warm						Cool					
Sex	Females			Males			Females			Males			Females			Males		
DV	Seg#	mean	Alt mean	Seg#	mean	Alt mean	Seg#	mean	Alt mean	Seg#	mean	Alt mean	Seg#	mean	Alt mean	Seg#	mean	Alt mean
EE	1	0.242	0.242	1	0.300	0.300	1	0.170	0.170	1	0.186	0.186	1	0.306	-	1	0.313	-
	2	0.163	X	2	0.207	X	2	0.106	X	2	0.102	X	2	0.212	-	2	0.212	-
	3	0.108	0.147	3	0.133	0.172	3	0.095	0.100	3	0.091	0.094	-	-	-	-	-	-
	4	0.080	0.080	4	0.093	0.093	-	-	-	-	-	-	-	-	-	-	-	-
	5	0.102	X	5	0.18	0.180	-	-	-	-	-	-	-	-	-	-	-	-
	6	0.170	0.141	-	-		-	-	-	-	-	-	-	-	-	-	-	-
RQ	1	0.871	-	1	0.849	-	1	0.874	-	1	0.925	-	1	0.914	-	1	0.907	-
	2	1.051	-	2	0.91	-	2	0.885	-	2	0.961	-	2	0.893	-	-	-	-
	-	-	-	3	1.081	-	-	-	-	-	-	-	-	-	-	-	-	-
	-	-	-	4	0.985	-	-	-	-	-	-	-	-	-	-	-	-	-
	-	-	-	5	0.915	-	-	-	-	-	-	-	-	-	-	-	-	-
RWL	1	0.108	-	1	0.112	-	1	0.223	-	1	0.237	0.237	1	0.192	0.192	1	0.188	-
	2	0.209	-	2	0.163	-	2	0.156	-	2	0.151	X	2	0.126	X	2	0.115	-
	3	0.129	-	3	0.280	-	-	-	-	3	0.128	0.133	3	0.105	0.117	-	-	-
	-	-	-	4	0.199	-	-	-	-	-	-	-	-	-	-	-	-	-
	-	-	-	5	0.157	-	-	-	-	-	-	-	-	-	-	-	-	-
VCO_2	1	0.709	0.709	1	0.873	0.873	1	0.505	0.505	1	0.496	X	1	0.944	-	1	0.958	-
	2	0.479	X	2	0.618	X	2	0.340	X	2	0.632	0.591	2	0.639	-	2	0.640	-
	3	0.341	0.438	3	0.412	0.518	3	0.278	0.314	3	0.331	0.331	-	-	-	-	-	-
	4	0.270	0.270	4	0.308	0.308	-	-	-	4	0.292	0.292	-	-	-	-	-	-
	5	0.337	X	5	0.567	0.567	-	-	-	-	-	-	-	-	-	-	-	-
	6	0.520	0.436	-	-	-	-	-	-	-	-	-	-	-	-	-	-	-
VO_2	1	0.813	0.813	1	1.022	1.022	1	0.577	0.577	1	0.623	0.186	1	1.033	1.033	1	1.055	-
	2	0.524	X	2	0.703	X	2	0.359	X	2	0.345	X	2	0.718	0.718	2	0.725	-
	3	0.363	0.467	3	0.448	0.580	3	0.322	0.338	3	0.304	0.094	3	1.083	X	-	-	-
	4	0.262	0.262	4	0.308	0.308	-	-	-	-	-	-	-	-	-	-	-	-
	5	0.337	X	5	0.605	0.605	-	-	-	-	-	-	-	-	-	-	-	-
	6	0.567	0.471	-	-	-	-	-	-	-	-	-	-	-	-	-	-	-

Table S4. Bayesian change point estimates for each experiment (Exp.: Variable, Warm, Cool), sex, and dependent variable (DV) across a 24 hour cycle. *Italics* indicate multiple change points located within 5 minutes of each other which are herein considered as a single change point. Dark grey columns indicate hours for which no change points were detected within an experiment.

			Time (hr:min)																	
Exp.	Sex	DV	1	2	3-4	5	6	7	8	9	10	11	12	13-18	19	20	21	22	23	24
Variable	Females	EE				*5:08, 5:26/5:28*									19:18	*20:12 /20:20*				
		RQ					6:40						*12:52 /12:53*		19:52					
		H_2O					*6:50 /6:55*	7:42			10:16									
		VCO_2				5:08									19:18					
		VO_2				*5:08,* ***5:26/5:28***	*6:51 /6:52*									*20:12 /20:20*				
	Males	EE					6:25								19:18	20:12	21:26			
		RQ					6:53		8:46											
		H_2O										11:00								
		VCO_2					6:25									*20:04 /20:12*	21:26			
		VO_2					*6:22 /6:25*								19:18	20:12	21:26			
Warm	Females	EE				5:16		*7:33 /7:34*								20:55				
		RQ																		
		H_2O																		
		VCO_2				5:16		7:34								20:55		22:18		
		VO_2				5:16		*7:33 /7:34*								20:55				
	Males	EE					6:48											*22:24 /22:26*		
		RQ							8:25											
		H_2O					*6:57*	7:28									21:36			
		VCO_2					6:48											*22:45 /22:48*		
		VO_2					6:48											*22:45 /22:48*		
Cool	Females	EE				5:16		*7:33 /7:34*								20:55				
		RQ																		
		H_2O																		
		VCO_2				5:16														
		VO_2				5:16		*7:33 /7:34*								20:55				
	Males	EE		2:22		5:56													*23:24 /23:26*	
		RQ																		
		H_2O																		
		VCO_2		*2:22/2:23 /2:27*		5:56									19:54				*23:24 /23:26*	
		VO_2		*2:22 / 2:54*		5:56													*23:34 / 23:36*	

Journal of Experimental Biology • Supplementary information
